# Interspecific Aggressions between Crested Porcupines and Roe Deer

**DOI:** 10.3390/ani10040623

**Published:** 2020-04-04

**Authors:** Lorenzo Lazzeri, Caterina Senini, Emiliano Mori

**Affiliations:** 1 Research Unit in Behavioural Ecology and Wildlife Management, Department of Life Sciences, University of Siena, Via P.A. Mattioli 4, 53100 Siena, Italy; lazzerilorenzo12@gmail.com; 2 Department of Veterinary Medicine Sciences, University of Bologna, Via Tolara di Sopra, 43, 40064 Ozzano dell’Emilia (Bologna), Italy; caterina.senini@gmail.com

**Keywords:** aggressions, behavioral interference, feeding sites, *Capreolus capreolus*, *Hystrix cristata*, open areas

## Abstract

**Simple Summary:**

Behavioral interspecific interference species is well-documented only amongst carnivore mammals, whereas being rare for ungulates and rodents. We report data on interactions between roe deer and crested porcupine at feeding sites. Aggressions by crested porcupines toward roe deer, often through chasing and more rarely through biting and attacking, were noted in about 16% of observations. In the remaining 84% of observations, roe deer and porcupines feed within the same feeding site, without interacting. Aggressions occurred mostly in warm months, when interspecific competition for food between these species is suggested to be the highest.

**Abstract:**

Despite being common amongst carnivore mammals, behavioral interference between wild herbivore species is poorly documented. Particularly, in temperate areas, where the ungulate guild is composed of a few species, and large-sized rodents are scarce, most cases of interspecific interactions involve at least one alien species. In this work, we report the first data on behavioral interactions between roe deer, *Capreolus capreolus*, and crested porcupine, *Hystrix cristata*. Aggressions by crested porcupines toward roe deer were observed in 34 out of 202 observations of both species feeding at the same site. In the other 168 observations, roe deer and porcupines shared the same feeding area, without any interaction. In 58% cases of interaction, porcupines chased and pushed roe deer away from feeding areas, and in several other cases, roe deer were bitten, or injured with quills. Aggressions by porcupines occurred mostly during warm months, when roe deer are mostly solitary and when competition for food between these species is suggested to be the highest, and against single female individuals.

## 1. Introduction

Interspecific and intraspecific interactions are particularly attractive for wildlife photographers and watchers [1,2,3,4,5], as they are often spectacular and, thus, easy to detect by non-specialists [6,7]. Behavioral interference is a form of interspecific interaction which may occur through direct physical aggression and/or resource depletion by the upper competitor toward the lower one [8,9,10,11]. Large-sized or gregarious species may outcompete small-sized or solitary ones for feeding sites [12,13]. Interspecific aggressive behavior is common for carnivores [14,15], whereas it is less documented for herbivores [16,17]. Amongst herbivore species, most interspecific aggressive interactions in temperate areas occur when at least one of the interacting species is alien [16,17,18]. Therefore, redefinition of species-distribution range due to human-mediated introductions and climatic change may provoke new interspecific encounters. 

For instance, the roe deer, *Capreolus capreolus*, is the most widespread and abundant native cervid species in Europe. In detail, in Italy, this species is recorded throughout the Alps and along the Apennine ridge, up to the southernmost peninsular regions, and its range is increasing [7,19]. The crested porcupine, *Hystrix cristata*, is the largest rodent in Italy [19], living in a number of habitat types, from woodlands to rural environments and to suburban areas [20,21]. Genetics [22], paleontology [23], reproductive phenology [24], behavioral ecology [25] and ectoparasitology [26] strongly suggest a North African origin of the Italian population of this species, which may have occurred after the Fall of the Western Roman Empire (but see also [27,28] for skull morphometrics). The crested porcupine may have evolved in the Pleistocene African savannah, where the ungulate guild was very rich in species [29,30,31]. During the last 50 years, this species has undergone a remarkable population increase and range expansion in Italy [32].

The diet of the crested porcupines in Central Italy depends on seasonal availability of trophic resources [21]. In cold months, porcupines mainly feed on underground vegetal organs (bulbs, tubers and roots), whereas a preference for stems, fruits and epigeal plant parts is shown during spring and summer [21,33,34]. Therefore, in warm months, porcupines may compete for food with native ungulates feeding in open areas, e.g., the roe deer, also in Italy [35,36,37]. The defense strategies by the crested porcupine toward potential predators and competitors include four displays, characterized by increasing aggressiveness [38,39]: (i) dorsal quill and crest erection (which is sufficient for over 50% of interactions); (ii) tail rattling through rattle quills; (iii) hind-foot stamping and growling; and (iv) backyard/sideways attack. Most times, predators desist, remaining uninjured, but they may also be wounded to death [38,39]. The same behavior may be displayed by crested porcupines toward conspecifics in captivity [40] or potential competitors, e.g., for food. This is particularly evident in warm months and at the start of autumn, i.e., when porcupines feed on epigeal parts of plants and on fruits [21,33].

The aim of this work was to describe interspecific interactions occurring between roe deer and crested porcupines at feeding sites (i.e., fallows and ecotones). We expected that most direct interactions would occur in warm months (spring and summer), i.e., when diet overlap amongst these species is likely to be the highest [33,37].

## 2. Materials and Methods

Field data on interactions between roe deer and crested porcupines in open areas (fallows and ecotones) were collected directly by 48 qualified selective hunters involved in roe deer culling from huts and turrets, as well as through the help of the local Hunting Agency (ATC Gr6). We asked hunters to collect data throughout the year, between 2015 and 2020, in 18 sites of Northern–Central Italy (provinces of Grosseto, Siena, Arezzo, La Spezia and Genoa). In this area, roe deer culling occurs between the 1st of October and the 15th of March. However, monitoring of roe deer occurrence is carried out by cullers throughout the year; consistently, observations (anecdotal data) occurred throughout the four seasons. In Tuscany, selective ungulate culling requires that interested hunters attend a 48-hour course to learn how to distinguish between ungulate species (roe deer, fallow deer (*Dama dama*), red deer (*Cervus elaphus*), mouflon (*Ovis aries*) and wild boar), as well as individual sex and age. Observations were carried out from vantage points located at least at 150–300 m of distance, always at dusk or dawn. Between 2018 and 2020, we filled a dataset including all the observations reported by asking notes to hunters: incomplete records (i.e., those lacking date or other information were not included in our analyses. Photographic data (*n* = 2) were also provided by other national mammal experts (A. Pastorino and A. Pieragnoli).

We filled a dataset with the following information: (1) location and date of observations; (2) season; (3) number of individuals of crested porcupines and roe deer; (4) roe deer sex and age (adult, i.e., ≥2 years old, or juveniles ≤2 years old, following body size, [7]); and (5) description of the interaction. Porcupines are sexually monomorphic: therefore, it was not possible to determine their sex without a direct manipulation [41]. We kept only records including all the required information; unluckily, it was not possible to obtain reliable data to distinguish between fawns and yearlings amongst juvenile roe deer. 

We used a generalized linear model, using a dataset with all observations indicating presence or absence of interspecific aggression (i.e., binomial regression model). We tested if the occurrence of an aggressive interaction between the roe deer and the crested porcupine was affected by season, cold months (October–March) vs. warm months (April–September), and by roe deer age (adult or juvenile) and sex (male or female). We ran the model through the R software (version 3.5.1., R Foundation for Statistical Computing, Wien, Austria).

## 3. Results

A total of 202 observations (94 in cold months, 108 in warm ones) with crested porcupines and roe deer (90 males, 102 females; 96 adults, 106 juveniles), feeding at a distance of 5–30 meters, were carried out at 18 sites located in Northern–Central Italy (total hours of observation = 171). Only 34 (16.8% cases) involved interspecific interactions between roe deer and crested porcupine, with the latter species always resulting in being dominant (Table S1 in Supplementary File).

**Figure 1:**
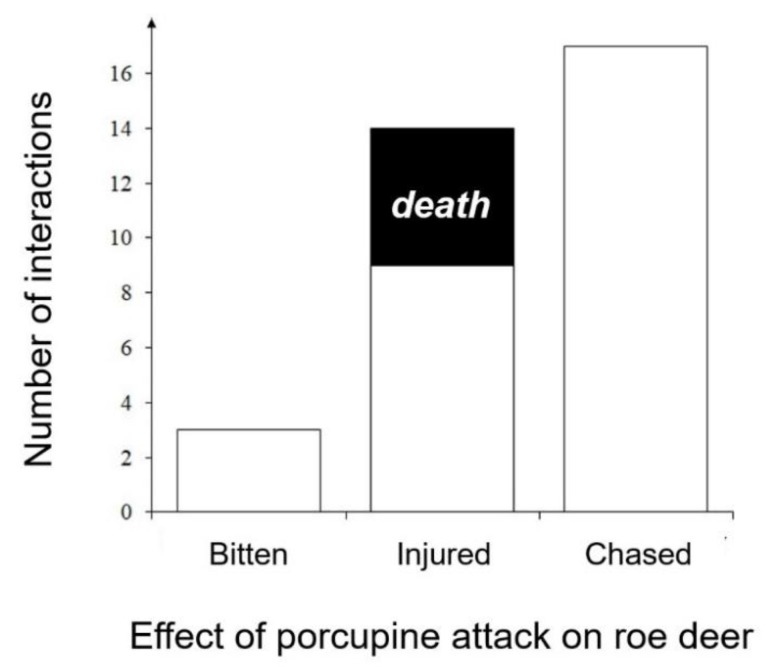
Aggressive interactions between crested porcupine and roe deer.

Most of the interactions (58.6%) were represented by chasing of crested porcupines toward one or two roe deer, whereas 31.0% involved roe deer getting injured with quills (resulting in five death cases) and 10.4% being bitten (Figure 1).

Aggressions were more likely to occur during the warm months (warm–cold months: 1.02 ± 0.13, *z* = 8.22, *p* < 0.001), against females (females–males: 3.84 ± 0.51, *z* = 10.02, *p* < 0.001) and juvenile roe deer (juvenile–adult: 3.35 ± 0.64, *z* = 9.45, *p* < 0.001).

## 4. Discussion

In this study, we reported, for the first time, some patterns of direct aggressive interactions between a large rodent and a cervid, i.e., the roe deer. Porcupines become particularly intolerant toward other species and conspecifics in cold months, i.e., at the peak of their reproductive period, both in feeding areas and in the surroundings of their burrows [42,43,44]. In cold months, harassments by crested porcupines (quill/crest erection) have been observed against wild boar, *Sus scrofa*, in feeding areas, and, in a few cases, wild boars have been wounded by porcupine quills (e.g., in Alta Val d’Elsa, A. Pieragnoli, personal communication 2020, [38]). Porcupines tend to avoid areas characterized by high densities of wild boar, particularly where rooting disturbance is high [42,45]. Furthermore, winter is the only period in which porcupines avoid den sharing with other species [43]. This may represent a sort of “defense” strategy toward feeding resources and offspring protection from potential predators and competitors [24,38,43,44].

Surprisingly, attacks by crested porcupines never occurred in cold months, i.e., when the roe deer mostly live in groups [46], and when most of the diet of the crested porcupine is composed by underground storage organs, which may limit interspecific competition [21,33,34]. In warm months, movements of the crested porcupines in Italy strongly increase, to reach open areas, where most food items consumed in these seasons occur [33,47,48,49]. In spring and summer, roe deer, particularly single females with juveniles, are quite widespread in open areas, which may enhance the potential for behavioral interference [50,51]. This is in line with observations on interference between fallow deer and roe deer, which increases in warm months, when open areas are attractive for both species [11,13,17]. In warm months, porcupines also use open areas for feeding, when cultivations provide this large rodent with clumped and abundant food [47,48,49]. In this context, encounters with female and juvenile roe deer may thus increase, thus creating the context for behavioral interactions. Accordingly, most physical attacks involving bites or injuries occurred toward females or young individuals, the latter having little or no experience. In general, aggressions require energy loss, which may explain why porcupines attacked roe deer only rarely [52], as already observed for fallow and roe deer [13]. Conversely, roe deer never displaced crested porcupine from feeding sites, despite being heavier. 

The collection of opportunistic and haphazard data is subject to many unknown patterns, due to how records arise. Particularly, our work involved woodland species active at night, when human sighting ability is the lowest. We cannot rule out that some other interactions may have occurred between roe deer and crested porcupine, without being recorded. Furthermore, information on recording efforts and biases cannot be evaluated when data are not collected through standardized protocols; thus, the use of citizen science should be circumscribed to a few kinds of scientific works, such as large-scale surveys or preliminary studies [53,54].

## 5. Conclusions

In an evolutionary timescale, we suggest that competition and predation pressure may have led crested porcupines living in open habitats in Africa to develop a quill armor and to feed mostly on underground storage organs, to limit competition with native grazer herbivores [31,55]. Where niche overlap increases, crested porcupines start to defend resources even through direct attacks [38,43,55], and this may happen both at den sites and at feeding sites, particularly where the crested porcupine may be an introduced species, e.g., in Italy. Despite being rare, aggressions of roe deer by crested porcupine occur mostly in the warm months and toward young individuals, which may be the most prone to approach porcupines. Amongst mammals, ungulates and rodents represent the main herbivore groups at temperate latitudes. Therefore, interspecific interactions can be predicted, even with effects on rodent populations [56,57,58]. Up until now, most studies on rodent–ungulate interactions have been conducted on small rodents, with very few studies involving large ones [42,45]. Large-sized rodents would require a higher food amount with respect to small-sized ones, thus enhancing the potential for competition with other herbivores. Future studies on interactions between ungulate and rodent species should be carried out to focus on both exploitation and interference mechanisms of competition.

## Supplementary Materials

The following are available online at https://www.mdpi.com/2076-2615/10/4/623/s1, Table S1: Aggressive interactions between crested porcupine and roe deer in open areas.
