# Supplementary material for: Interspecific Aggressions between Crested Porcupines and Roe Deer
Source: Animals (Basel). 2020 Apr 4;10(4):623. doi: 10.3390/ani10040623 (PMC7222735; doi:10.3390/ani10040623)
Supplement: Supplementary file 1 [file animals-10-00623-s001.pdf]

Supplementary File

Table S1. Aggressive interactions between crested porcupine and roe deer in open areas.

ID	Date	Location	Season	Hour	Habitat Type	N° Porcupines	N° Roe Deer	Age & Sex of Roe Deer	Roe Deer exposure
1	20/06/2015	Prata (GR)	Summer	19:49:00	Open area	1	1	Juvenile male	Chased
2	28/08/2015	Orgia (SI)	Summer	21:35:00	Ecotone	2	2	Female and juvenile	Chased
3	30/08/2015	Pratovecchio (AR)	Summer	20:40:00	Ecotone	3	1	Adult female	Chased
4	17/05/2016	Arezzo (AR)	Spring	20:35:00	Open area	1	1	Juvenile male	Bitten
5	22/06/2016	Pratovecchio (AR)	Summer	20:39:00	Open area	3	1	Juvenile female	Injured with quills
6	03/08/2016	Passo della Consuma (AR)	Summer	20:23:00	Open area	1	1	Juvenile female	Chased
7	11/08/2016	Pitigliano (GR)	Summer	20:33:00	Ecotone	2	1	Adult female	Chased
8	11/08/2016	Sarzana (SP)	Summer	-	-	-	-	Juvenile male	Injured with quills and found dead
9	06/09/2016	Braccagni (GR)	Summer	19:50:00	Ecotone	2	1	Juvenile male	Chased
10	27/10/2016	Castell'Azzara (GR)	Autumn	21:42:00	Ecotone	2	2	Adult males	Chased
11	15/04/2017	Orgia (SI)	Spring	-	-	-	-	Juvenile male	Injured with quills and found dead
12	20/05/2017	Arezzo (AR)	Spring	20:37:00	Open area	2	1	Adult female	Chased
13	26/05/2017	Colombaio Quercegrossa (SI)	Spring	21:48:00	Open area	2	2	Adult females	Chased
14	05/06/2017	Sarzana (SP)	Summer	21:10:00	Open area	2	1	Juvenile female	Injured with quills
15	18/06/2017	Quarata (AR)	Summer	20:51:00	Ecotone	3	1	Juvenile female	Chased
16	08/07/2017	Scarlino Scalo (GR)	Summer	21:25:00	Open area	1	1	Juvenile female	Injured with quills
17	10/07/2017	Forcoli - Montieri (GR)	Summer	20:01:00	Open area	2	1	Juvenile female	Chased
18	09/09/2017	Quarata (AR)	Summer	20:54:00	Ecotone	1	1	Juvenile female	Bitten
19	19/09/2017	Boccheggiano (GR)	Summer	-	-	-	-	Juvenile female	Injured with quills and found dead
20	15/11/2017	Passo della Consuma (AR)	Autumn	20:27:00	Ecotone	1	1	Adult female	Injured with quills
21	04/05/2018	Monteroni d'Arbia (SI)	Spring	20:19:00	Ecotone	1	1	Juvenile female	Bitten
22	18/05/2018	Follonica (GR)	Spring	19:57:00	Open area	1	1	Juvenile female	Injured with quills
23	28/05/2018	Forcoli - Montieri (GR)	Spring	20:05:00	Open area	3	1	Juvenile male	Injured with quills
24	28/05/2018	Mele (GE)	Spring	06:25:00	-	-	-	Juvenile female	Injured with quills and found dead
25	22/11/2018	Asciano (SI)	Autumn	19:47:00	Ecotone	2	1	Adult female	Chased
26	11/05/2019	Orgia (SI)	Spring	21:29:00	Open area	1	2	Adult females	Chased

27	01/06/2019	Passo della Consuma (AR)	Spring	-	-	-	-	Juvenile male	Injured with quills and found dead
28	14/06/2019	Quarata (AR)	Spring	20:58:00	Open area	1	1	Adult male	Chased
29	07/07/2019	Pratovecchio (AR)	Summer	20:43:00	Ecotone	1	1	Adult female	Chased
30	15/09/2019	Forcoli - Montieri (GR)	Summer	20:07:00	Open area	2	1	Adult female	Chased
31	18/09/2019	Pratovecchio (AR)	Summer	20:48:00	Open area	1	1	Young female	Injured with quills
32	27/09/2019	Forcoli - Montieri (GR)	Autumn	20:09:00	Ecotone	2	1	Young female	Injured with quills
33	11/10/2019	Forcoli - Montieri (GR)	Autumn	20:14:00	Open area	2	1	Adult female	Chased
34	18/10/2019	Buonconvento (SI)	Autumn	19:53:00	Ecotone	3	1	Young male	Injured with quills
